# Hypermethylation in H3K9me3 regions characterizes the centenarian methylomes in healthy aging

**DOI:** 10.1093/nsr/nwad067

**Published:** 2023-03-09

**Authors:** Fu-Hui Xiao, Hao-Tian Wang, Xiao-Qiong Chen, Ming-Xia Ge, Dongjing Yan, Xing-Li Yang, Li-Qin Yang, Rong Lin, Rong-Hui Guo, Wen Zhang, Nelson Leung-Sang Tang, Yonghan He, Jumin Zhou, Wang-Wei Cai, Qing-Peng Kong

**Affiliations:** State Key Laboratory of Genetic Resources and Evolution, Key Laboratory of Healthy Aging Research of Yunnan Province, Kunming Key Laboratory of Healthy Aging Study, Kunming Institute of Zoology, Chinese Academy of Sciences, China; KIZ/CUHK Joint Laboratory of Bioresources and Molecular Research in Common Diseases, China; State Key Laboratory of Genetic Resources and Evolution, Key Laboratory of Healthy Aging Research of Yunnan Province, Kunming Key Laboratory of Healthy Aging Study, Kunming Institute of Zoology, Chinese Academy of Sciences, China; KIZ/CUHK Joint Laboratory of Bioresources and Molecular Research in Common Diseases, China; Kunming College of Life Science, University of Chinese Academy of Sciences, China; State Key Laboratory of Genetic Resources and Evolution, Key Laboratory of Healthy Aging Research of Yunnan Province, Kunming Key Laboratory of Healthy Aging Study, Kunming Institute of Zoology, Chinese Academy of Sciences, China; State Key Laboratory of Genetic Resources and Evolution, Key Laboratory of Healthy Aging Research of Yunnan Province, Kunming Key Laboratory of Healthy Aging Study, Kunming Institute of Zoology, Chinese Academy of Sciences, China; KIZ/CUHK Joint Laboratory of Bioresources and Molecular Research in Common Diseases, China; Kunming College of Life Science, University of Chinese Academy of Sciences, China; Department of Biochemistry and Molecular Biology, Hainan Medical College, China; State Key Laboratory of Genetic Resources and Evolution, Key Laboratory of Healthy Aging Research of Yunnan Province, Kunming Key Laboratory of Healthy Aging Study, Kunming Institute of Zoology, Chinese Academy of Sciences, China; State Key Laboratory of Genetic Resources and Evolution, Key Laboratory of Healthy Aging Research of Yunnan Province, Kunming Key Laboratory of Healthy Aging Study, Kunming Institute of Zoology, Chinese Academy of Sciences, China; KIZ/CUHK Joint Laboratory of Bioresources and Molecular Research in Common Diseases, China; Department of Biology, Hainan Medical College, China; State Key Laboratory of Genetic Resources and Evolution, Key Laboratory of Healthy Aging Research of Yunnan Province, Kunming Key Laboratory of Healthy Aging Study, Kunming Institute of Zoology, Chinese Academy of Sciences, China; KIZ/CUHK Joint Laboratory of Bioresources and Molecular Research in Common Diseases, China; Kunming College of Life Science, University of Chinese Academy of Sciences, China; Department of Biochemistry and Molecular Biology, Hainan Medical College, China; KIZ/CUHK Joint Laboratory of Bioresources and Molecular Research in Common Diseases, China; Department of Chemical Pathology and Laboratory for Genetics of Disease Susceptibility, Li Ka Shing Institute of Health Sciences, and School of Biomedical Sciences, Faculty of Medicine, The Chinese University of Hong Kong, China; State Key Laboratory of Genetic Resources and Evolution, Key Laboratory of Healthy Aging Research of Yunnan Province, Kunming Key Laboratory of Healthy Aging Study, Kunming Institute of Zoology, Chinese Academy of Sciences, China; Key Laboratory of Animal Models and Human Disease Mechanisms of the Chinese Academy of Sciences, Key Laboratory of Healthy Aging Research of Yunnan Province, Kunming Institute of Zoology, China; Department of Biochemistry and Molecular Biology, Hainan Medical College, China; State Key Laboratory of Genetic Resources and Evolution, Key Laboratory of Healthy Aging Research of Yunnan Province, Kunming Key Laboratory of Healthy Aging Study, Kunming Institute of Zoology, Chinese Academy of Sciences, China; CAS Center for Excellence in Animal Evolution and Genetics, Chinese Academy of Sciences, China; KIZ/CUHK Joint Laboratory of Bioresources and Molecular Research in Common Diseases, China

Centenarians, as excellent examples of extreme yet successful aging, not only display a much longer lifespan but can also delay or even escape major age-related diseases, such as cardiovascular disorders, neurodegenerative diseases, and cancer [1]. Studies have suggested that genetic factors account for 20%–30% of human longevity [2]; however, only a handful of longevity-associated genes/mutations have been observed in long-lived individuals, even with the use of genome-wide scanning/sequencing assays [3,4]. In contrast, evidence has shown the crucial roles of epigenetic alternations in aging and age-related diseases [5], suggesting that epigenetic modifications play central roles in human healthy aging. Thus, a comprehensive analysis of the epigenomic landscape of centenarians is urgently needed.

To date, however, our current knowledge of centenarian DNA methylation (DNAm) modification is primarily based on Illumina Infinium HumanMethylation450 BeadChip [6], which only covers ∼450 000 CpG sites located in well-studied regulatory regions (e.g. promoter regions). Such results may bias our understanding as increasing evidence supports the functional potential of gene bodies and intergenic and intronic regions [7]. Screening centenarian DNAm across whole genomes with higher resolution and coverage is thus of help in determining the methylation loci involved in healthy aging. Thus far, the DNAm map at the base level has only been obtained for a single Caucasian centenarian individual [8], making it difficult to reveal the common epigenetic characteristics in long-lived people. Therefore, decoding DNA methylomes at base-resolution in a centenarian cohort is essential to decipher the epigenetic regulatory patterns of healthy and extreme aging in humans.

To construct a single-base resolution DNAm map of centenarians, we performed whole-genome bisulfite sequencing (WGBS) of peripheral blood samples from 111 individuals from Hainan Province, China, including 57 centenarians (age: 102.2 ± 2.6 years old), 22 elderly (age: 74.0 ± 2.7 years old) and 32 younger (age: 57.9 ± 5.0 years old) F1SP samples (i.e. spouses of centenarian-children; treated as controls here) (Fig. 1A and Supplementary Fig. S1). We obtained and analyzed 23 617 367 CpG sites covered by over 60% of the 111 samples (read coverage ≥10), representing ∼84.35% of CpG sites across whole genome (Supplementary Fig. S2A and B). Then, three outlier samples were removed from subsequent analyses (Supplementary Fig. S2C). Results showed that centenarians only exhibited a very slight global DNAm loss (Fig. 1B).

**Figure 1.**
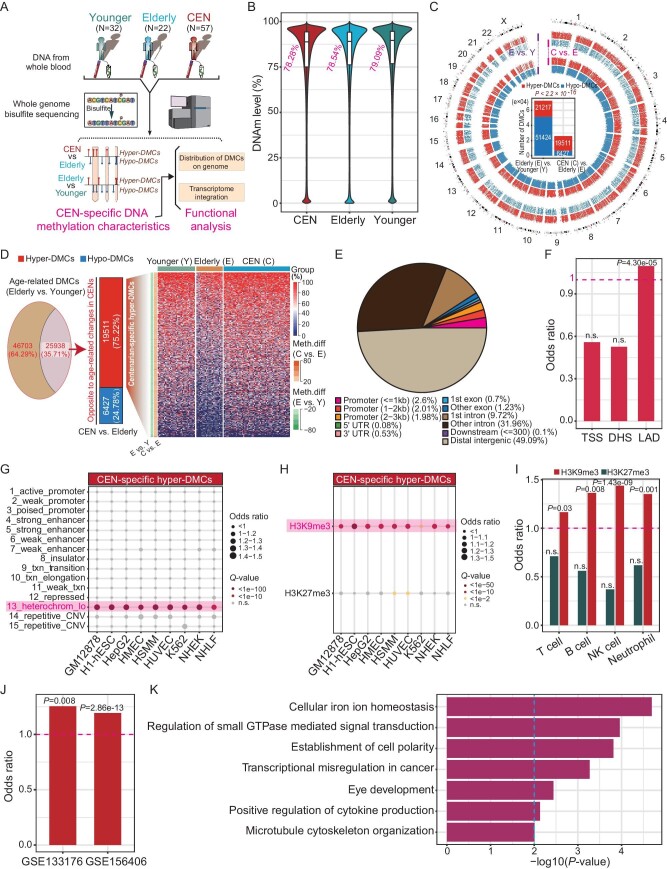
A unique hypermethylation pattern in the constitute heterochromatic regions across centenarians’ epigenome. (A) Diagram of the study design. (B) Overall methylation differences between CENs, elderly and younger controls. In the overall methylation level analysis, the methylation level of a CpG site was represented by the average *β*-value of the samples in one group. (C) Circos plot for the distribution of age-related DMCs between the elderly and younger controls (labelled as ‘E vs. Y’) and those DMCs with methylation differences opposite to the age-related changes in the CENs (i.e. CEN vs. elderly control group; labelled as ‘C vs. E’). A higher-than-expected fraction of hyper-DMCs existed in the CENs. (D) Heatmap plot for the methylation levels of the 19 511 CEN-specific hyper-DMCs. (E) Most CEN-specific hyper-DMCs located in distal intergenic and intronic regions. (F) Enrichment analysis revealed that the CEN-specific hyper-DMCs were not overrepresented in TSS nearby regions (−3000 ∼ +3000 bp; defined as promoter regions) and DHSs but overrepresented in LADs on the genome. (G) Enrichment analysis showed that the CEN-specific hyper-DMCs were enriched in heterochromatic regions. (H) CEN-specific hyper-DMCs overrepresented in H3K9me3 regions rather than in H3K27me3 regions. (I) CEN-specific hyper-DMCs overrepresented in H3K9me3 regions instead of H3K27me3 regions in different blood cell types, including T cell, B cell, natural killer (NK) cell, and neutrophil. (J) CEN-specific hyper-DMCs overrepresented in genomic regions with H3K9me3 loss in senescent cells (GSE133176, CSB-deficient induced senescent fibroblasts; GSE156406, senescent human mesenchymal stem cells). (K) Biological function enrichments for genes repressed by the CEN-specific hyper-DMCs located in the nearby H3K9me3 regions. (n.s., non-significant).

Next, we attempted to decipher the specific methylation characteristics of centenarians across the whole genome at single-base level. Using a threshold of >10% methylation difference and *Q*-value <0.01, we identified 72 641 differentially methylated CpG sites (DMCs) between the elderly and younger control samples (considered as age-related DMCs), including 21 217 hyper-DMCs and 51 424 hypo-DMCs (Fig. 1C). We then asked whether some of these age-related DMCs exhibited a different direction of methylation changes in the centenarians from that associated with age, say, for example, some DMCs display hypomethylation with age but keep hypermethylated in centenarians, or vice versa. Indeed, of the 72 641 age-related DMCs, we found that 25 938 (35.71%) exhibited methylation differences in the centenarians (i.e. centenarians vs. elderly control samples) but with methylation direction opposite to the age-related changes (Fig. 1C and D). Specifically, 19 511 (75.22%) out of the 25 938 DMCs were hypermethylated in the centenarians (compared with the elderly controls) but hypomethylated with age (Fig. 1C and D). These findings suggest that some CpG sites remain hypermethylated in centenarians, which however exhibit a gradual loss of methylation with age and thus are hypomethylated in the elderly individuals

(when compared with the younger controls).

Furthermore, we showed that these hyper-DMCs had a median methylation difference of 15.84%, with nearly three-quarters (72.78%) being intermediately methylated (Supplementary Fig. S3). This ratio was significantly higher than the expected value (16.75%) from genomic background (Supplementary Fig. S3), suggesting that this methylation pattern is unlikely to be a random process and may play a role in genome regulation. We also found that the hyper-DMCs had a relatively high probability of being located at a closer distance to the longevity-related variants (LRVs) compared to the other covered CpG sites (Supplementary Fig. S4), suggesting that the hyper-DMCs, or some of them, might have potential associations with human healthy aging and longevity.

We then explored the distribution pattern of these 19 511 centenarian-specific hyper-DMCs and found that 6.59% of hyper-DMCs were located in promoter regions (−3000 to +3000 bp from the transcription start sites (TSSs)), whereas 49.09% and 41.68% were located in distal intergenic and intronic regions, respectively (Fig. 1E). Enrichment analysis showed that there was no enrichment for hyper-DMCs in the promoter regions (Fig. 1F). In addition, the hyper-DMCs were not overrepresented in DNase I hypersensitive sites (DHSs), a feature of ‘open chromatin’, in GM12878 cells (lymphoblastoid cell line) (Fig. 1F). In contrast, the hyper-DMCs displayed an overrepresentation in lamina-associated domains (LADs) (Fig. 1F). These findings suggest that the centenarian-specific hyper-DMCs are preferentially located in TSS distal regions or inactive regions on the genome.

To further investigate the biological implication of the centenarian-specific hyper-DMCs, we investigated the distribution of the hyper-DMCs in different chromatin states across the genome. According to the annotation of the standard 15 chromatin states in B-lymphoblastoid cells (GM12878) using the ChromHMM model, we discovered that the hyper-DMCs were mainly enriched in heterochromatic regions (Fig. 1G). A similar pattern was also observed in other cell types, including mammary epithelial cells (HMEC), normal lung fibroblasts (NHLF), etc. (Fig. 1G). Since heterochromatin loss is an important cause of aging [1], these results suggest that the centenarian-specific hyper-DMCs are likely involved in the control of the heterochromatin stabilization across the genome.

We then explored the distribution of the centenarian-specific hyper-DMCs in the constitutive and facultative heterochromatic regions (marked by H3K9me3 and H3K27me3, respectively) in GM12878 cells. Interestingly, results showed that the hyper-DMCs were significantly overrepresented in the H3K9me3 regions rather than the H3K27me3 regions (Fig. 1H). Likewise, this observation was also repeated in other cell types, such as HMEC and NHLF (Fig. 1H). To confirm this finding, we collected the H3K9me3 peak information of four blood cell types (i.e. T cell, B cell, natural killer cell, neutrophil) and found that the hyper-DMCs were overrepresented in H3K9me3 regions (Fig. 1I). We also obtained the annotation of genomic regions with H3K9me3 loss in senescent cells, and observed that the hyper-DMCs were overrepresented in these regions (Fig. 1J). Meanwhile, we showed that these hyper-DMCs exhibited no overrepresentation in H3K27ac and CTCF regions in GM12878 cells (Supplementary Fig. S5). We performed enrichment analysis for the DMCs satisfying the absolute methylation difference of >20%. Results showed that these hyper-DMCs were still significantly overrepresented in H3K9me3 regions annotated in GM12878 cells (Supplementary Fig. S6). We then evaluated the expression of TEs in the H3K9me3 regions (GM12878) with hyper-DMCs using the RNA-seq data of the same batch of samples, and found that 98.95% (280/283) of differentially expressed TEs were downregulated in the centenarians compared to the elderly controls, who displayed a trend of TE upregulation corresponding to the younger controls (Supplementary Fig. S7A and B). Taken together, these findings indicate that the centenarian-specific hyper-DMCs have some contributions in alleviating the loss of constitutive heterochromatin during aging.

To explore the potential function of these centenarian-specific hyper-DMCs in H3K9me3 regions annotated in GM12878 cells, we focused on the expression of genes with their promoter regions overlapped by the 337 H3K9me3 regions carrying at least 3 hyper-DMCs. Among the 238 detected genes that overlap with these regions, 83 displayed significant expression differences between the centenarians and the elderly controls (*P* < 0.05), a considerable proportion (60.24%, 50/83) of which were downregulated in the centenarians. Results showed that 24 of the 50 downregulated genes were zinc finger (ZNF) protein-coding genes. Here we performed enrichment analysis for the remaining 26 genes, and found that they were significantly enriched in biological processes or pathways including cellular iron ion homeostasis (GO:0006879), regulation of small GTPase mediated signal transduction (GO:0051056), transcriptional misregulation in cancer (hsa05202), positive regulation of cytokine production (GO:0001819) (*P* < 0.01) (Fig. 1K). Several genes in these pathways (e.g. *MYC* and *FXN*) and in ZNF family (e.g. *ZNF10* and *ZNF268*) have close associations between their downregulation and healthy aging and longevity (Supplementary Fig. S8A–C). For example, the gene *MYC* proto-oncogene is a tumor invasion and metastasis associated regulator, whose downregulation has been reported to improve longevity and health span in mice [9]. Thus, the hyper-DMCs located in the nearby H3K9me3 region of *MYC* may contribute to healthy aging via suppressing its expression (Supplementary Fig. S8A). An additional case comes from *ZNF10*, which has a function in promoting cancer progression [10], it is likely that the hyper-DMCs in H3K9me3 regions nearby *ZNF10* has potential to contribute to human healthy aging by inhibiting tumorigenesis (Supplementary Fig. S8B).

Taken together, our study constructs a comprehensive DNAm map of a Chinese centenarian cohort and, importantly, identifies plenty of centenarian-specific hyper-DMCs in H3K9me3 regions, which is different from the signal associated with age and most likely contributes to healthy human aging and longevity through stabilizing constitute heterochromatin. Our work thus provides some novel insights into the epigenetic basis of health-protection in longevity people.

## DATA AVAILABILITY

The high-throughput sequencing data reported in this paper have been deposited in the Genome Sequence Archive in National Genomics Data Center, China National Center for Bioinformation/Beijing Institute of Genomics, Chinese Academy of Sciences, under accession number HRA000502 and HRA003301 that are publicly accessible at https://bigd.big.ac.cn/gsa. All codes are available upon request.

## Supplementary Material

nwad067_Supplemental_File
