# Supplementary material for: Hypermethylation in H3K9me3 regions characterizes the centenarian methylomes in healthy aging
Source: Natl Sci Rev. 2023 Mar 9;10(6):nwad067. doi: 10.1093/nsr/nwad067 (PMC10171629; doi:10.1093/nsr/nwad067)
Supplement: nwad067_Supplemental_File [file nwad067_supplemental_file.doc]

**Supplementary information for**

**Hypermethylation in H3K9me3 regions characterizes the centenarian methylomes in healthy aging**

Fu-Hui Xiao^1,4,#^, Hao-Tian Wang^1,4,5,#^, Xiao-Qiong Chen^1^, Ming-Xia Ge^1,4,5^, Dongjing Yan^2^, Xing-Li Yang^1^, Li-Qin Yang^1,4^, Rong Lin^6^, Rong-Hui Guo^1,4,5^, Wen Zhang^2^, Nelson Leung-Sang Tang^4,7^, Yonghan He^1^, Jumin Zhou^8^, Wang-Wei Cai^2,*^, Qing-Peng Kong^1,3,4,*^

^1^ State Key Laboratory of Genetic Resources and Evolution, Key Laboratory of Healthy Aging Research of Yunnan Province, Kunming Key Laboratory of Healthy Aging Study, Kunming Institute of Zoology, Chinese Academy of Sciences, Kunming 650201, China;

^2^ Department of Biochemistry and Molecular Biology, Hainan Medical College, Haikou 571199, China;

^3^ CAS Center for Excellence in Animal Evolution and Genetics, Chinese Academy of Sciences, Kunming 650201, China;

^4^ KIZ/CUHK Joint Laboratory of Bioresources and Molecular Research in Common Diseases, Kunming 650201, China;

^5^ Kunming College of Life Science, University of Chinese Academy of Sciences, Beijing 100049, China;

^6^ Department of Biology, Hainan Medical College, Haikou 571199, China;

^7^ Department of Chemical Pathology and Laboratory for Genetics of Disease Susceptibility, Li Ka Shing Institute of Health Sciences, and School of Biomedical Sciences, Faculty of Medicine, The Chinese University of Hong Kong, Hong Kong, China;

^8^ Key Laboratory of Animal Models and Human Disease Mechanisms of the Chinese Academy of Sciences, Key Laboratory of Healthy Aging Research of Yunnan Province, Kunming Institute of Zoology, Kunming 650201, China.

^#^ These authors contributed equally.

* **Corresponding author:**

Qing-Peng Kong, State Key Laboratory of Genetic Resources and Evolution, Kunming Institute of Zoology, Chinese Academy of Sciences, Kunming 650201, China.

Telephone: +86-871-65199985; Fax: +86-871-65199985

E-mail: kongqp@mail.kiz.ac.cn (Qing-Peng Kong)

Wang-Wei Cai, Department of Biochemistry and Molecular Biology, Hainan Medical College, Haikou 571199, China.

Telephone: +86-898-66968753; Fax: +86-898-66893170

E-mail: [caiww591020@163.com](mailto:caiww591020@163.com) (Wang-Wei Cai)

**METHODS**

**Sampling, DNA extraction, and WGBS sequencing**

Peripheral blood samples were collected from centenarians, elderly and younger F1SPs in families from Hainan Province, China, as described in our prior study [1]. We selected the F1SP, who have no blood relationships with centenarians, as controls because the centenarian-children may inherit some epigenetic information being responsible for the longevity. Due to gender bias in the collected samples, we only considered female individuals for this study. We sampled a total of 111 individuals, including 57 centenarians, 22 elderly and 32 younger F1SPs for WGBS. Since the centenarian families are mostly located in remote villages in Hainan province, we could not do more elaborate examination as done in laboratory or hospital. Nevertheless, we still managed to assay the proportions of two major white cells (i.e., lymphocytes and granulocytes) in peripheral blood samples. All these samples have undergone RNA sequencing, as described in our prior study [1]. All research protocols were approved by the Ethics Committee at the Kunming Institute of Zoology, Chinese Academy of Sciences. Written informed consent was obtained from each participant before sampling.

Genomic DNA from peripheral blood cells was purified using an AxyPrepTM Blood Genomic DNA Maxiprep kit (Axygen) following the manufacturer’s instructions. A Qubit™ dsDNA High-Sensitivity Assay was used to quantify genomic DNA. For each sample, genomic DNA with 0.5% unmethylated Lambda DNA spike-in was sonicated into small fragments ranging from 100–300 bp using a Covaris S2 system. After DNA-end repair, 3’-dA overhang, and adapter ligation, bisulfite treatment was performed using an EZ DNA Methylation-Gold kit (Zymo Research) according to the manufacturer’s protocols. The DNA bisulfite conversion efficiency is approximately 99% in this study. Final qualified WGBS libraries were sequenced on an Illumina HiSeq platform in paired-end mode with 150 bp by the Beijing Genomics Institute, Shenzhen, China (<https://www.genomics.cn/>).

**Sequencing read alignment**

The raw reads were filtered using in-house script, based on the following conditions: 1) containing adaptors, 2) containing N > 10% (N represents base that could not be determined) and 3) the Qscore (Quality value) of over 10% of bases of the read is ≤ 20. Those sequence reads that passed the quality control were assessed by the FastQC program (http://www.bioinformatics.babraham.ac.uk/projects/fastqc). The clean reads with Q20 > 90% and Q30 > 85% were retained for subsequent analyses. The clean reads in pairs were then aligned to the NCBI (build 37/hg19) human genome using Bismark (v0.17.0) with default parameters (e.g., --minins 0, --maxins 500, --bowtie2) and bowtie2 (v2.1.0) for read alignment [2,3].

**Estimation of methylation level of CpG sites**

The methylation level of each CpG site was represented as a *β*-value, which was estimated from WGBS by the formula:


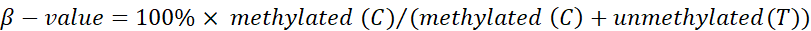


*β*-values range from 0% (completely unmethylated) to 100% (completely methylated). The *bismark_methylation_extractor* script in Bismark (v0.17.0) was used to extract the methylation call for the covered cytosine sites with the parameters: --CX --comprehensive --counts --bedGraph --cytosine_report [2].

**Differential methylation analysis**

The CpG sites with a minimum coverage of ten reads in over 60% of the 111 samples were retained for subsequent analyses. In this process, the sites with read-coverage of < 5 were considered as undetected. We then calculated the *β*-value of the sites and corrected the batch effect by the *removeBatchEffect* function in limma R package [4]. The final WGBS data object containing the information of read-coverage at each site was reconstructed from the batch-corrected *β*-value matrix using the *reconstruct* function in the methylKit Bioconductor package [5]. Although there were no significant differences in the detected cell types (i.e., lymphocytes and granulocytes) between the centenarian, elderly and younger F1SP groups, we still estimated the cell-composition of the samples to further reduce its effect on analyses. We obtained a covariate matrix of blood cell type compositions of samples using the *ReFACTor* program in GLINT with parameter: k = 5 (Supplementary Fig. S9) [6]. The *calculateDiffMeth* function in the methylKit Bioconductor package was used to identify DMCs, considering the cell-composition matrix as a covariate [5]. We defined DMCs using the thresholds of > 10% absolute methylation differences and *Q*-value < 0.01.

**Integrated analysis of methylomes and transcriptomes**

The RNA-seq data used in this study were previously submitted to the Genome Sequence Archive (GSA) in the BIG Data Center under accession number CRA000515 [1]. And part of RNA-seq data is available upon request. The *Combat_seq* function in the sva Bioconductor package was used to correct the batch effects of sampling batches and library types in the RNA-seq data [7]. Here, DESeq2 Bioconductor package was used to analyze gene expression differences between the two groups [8]. The expression level of each gene was represented by a vst-transformed read-count using the *vst* function in the DESeq2. In addition, the annotation information of TEs were obtained from the study of Kong et al. [9]. The UCSC liftOver tool was used to convert the TE annotation from hg38 to hg19 coordinates. We used the REdiscoverTE tool to quantify TEs’ expression levels [9], which were represented by Transcripts Per Million (TPM). The batch effects (i.e., sampling batches and library types) were corrected by *removeBatchEffect* in the limma package, which was used to perform the TE differential expression analysis [4].

**Gene annotation**

Build-matched (hg19) RefSeq gene annotation was downloaded from the UCSC database (https://genome.ucsc.edu/). The R/Bioconductor package ChIPseeker was used for gene annotation [10]. In addition, the standard 15 chromatin states and histone modification (e.g., H3K9me3, H3K27me3, CTCF) in the nine cell types were download from the ENCODE database (https://www.genome.ucsc.edu/ENCODE/). The DHS information in GM12878 and the peak information of H3K9me3 and H3K27me3 in four blood cell types (i.e., T cell, B cell, natural killer cell, neutrophil) were also downloaded from the ENCODE database (https://www.genome.ucsc.edu/ENCODE/). The LAD information was obtained through the UCSC Table Browser data retrieval tool (https://genome.ucsc.edu/cgi-bin/hgTables) with track “LaminB1 (Tig3)”. The annotation of genomic regions with H3K9me3 loss in senescent cells was collected from previous studies [11,12]. The LRVs were retrieved from the PheGenI and SNPedia databases [13,14].

**Enrichment analysis**

LOLA was used to conduct enrichment analysis for the DMCs in different genomic elements (e.g., histone modifications) , with Fisher’s exact test performed to calculate *P*-values [15]. Metascape was used to analyze the enriched Gene Ontology (GO) biological processes and Kyoto Encyclopedia of Genes and Genomes (KEGG) pathways based on a *P*-value cutoff of < 0.01 [16].

**Data visualization**

The distribution of the DMCs across the genome was drawn by Circos software. The PCA results were plotted by the R package factoextra.

**SUPPLEMENTARY FIGURES**


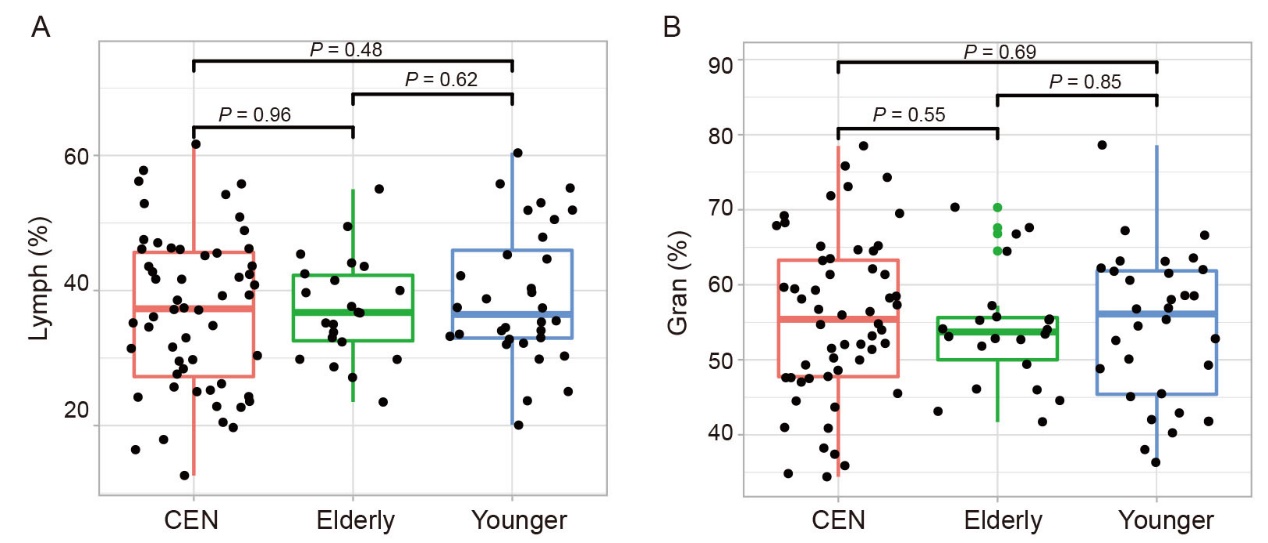


**Figure S1. Proportions of lymphocytes (A) and granulocytes (B) in centenarians, elderly and younger F1SPs.** The proportions of lymphocytes and granulocytes in peripheral blood samples had no significant difference between the centenarian, elderly and younger F1SP groups.


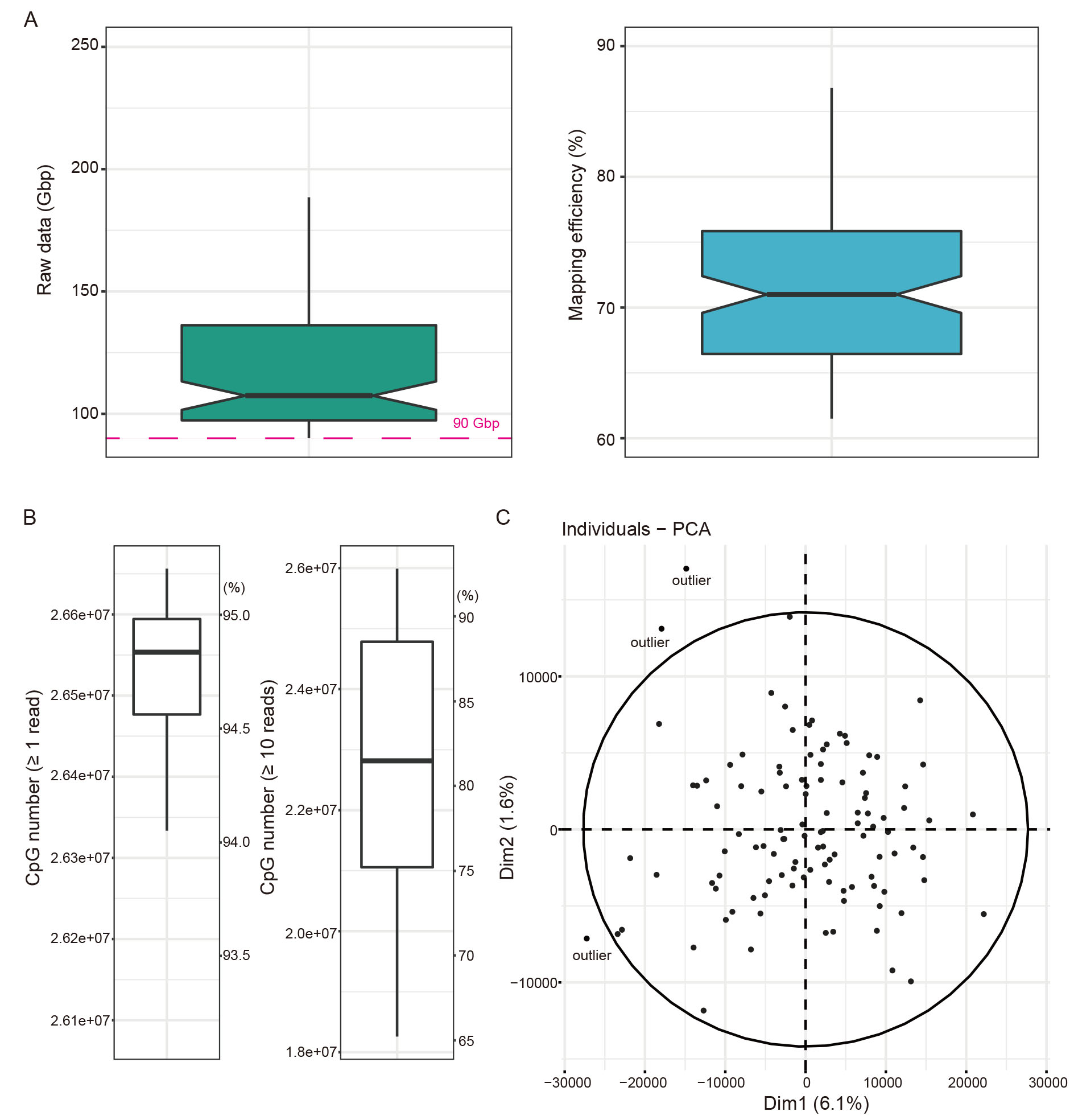


**Figure S2. Data summary of WGBS.** (**A**) Raw data of WGBS and mapping efficiency of data. **(B)** Covered CpG sites in the samples. **(C)** PCA analysis showed that three samples were considered as outliers (ellipse level = 0.98).


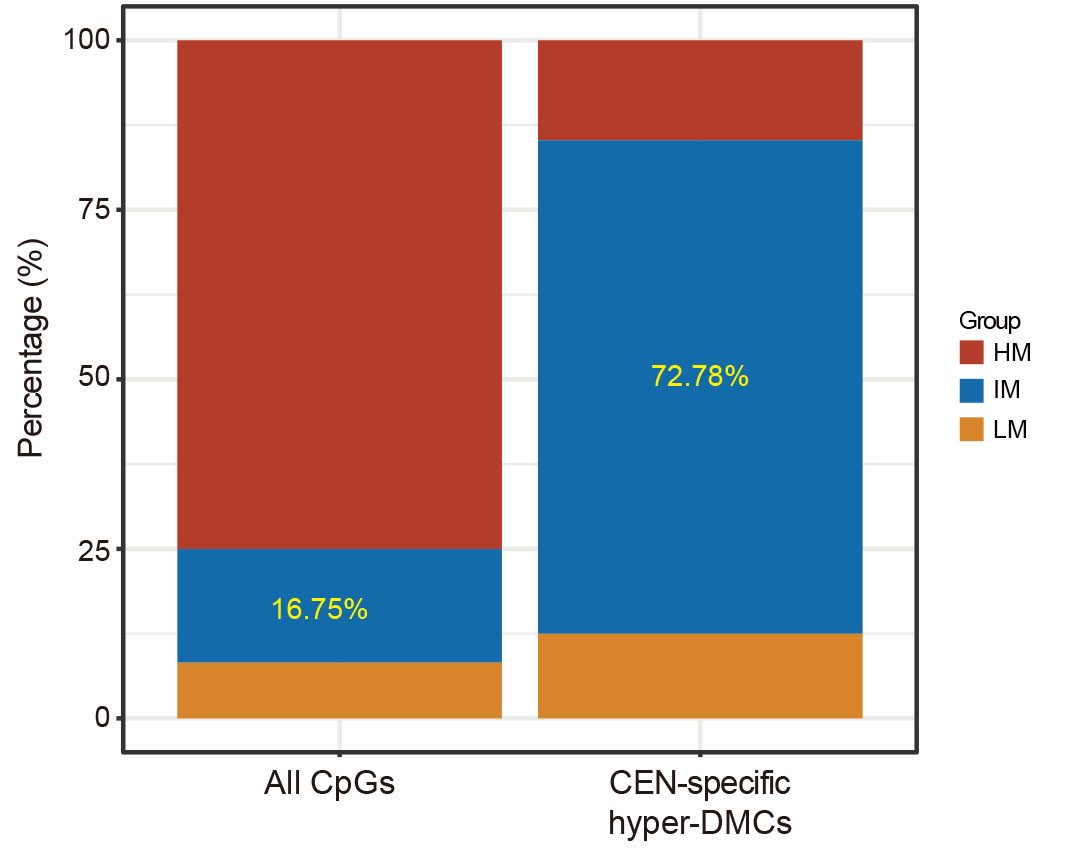


**Figure S3. Larger-than-expected number of CEN-specific hyper-DMCs were intermediately methylated (IM: 25%–75% methylation ratio; highly methylated (HM): > 75% methylation ratio; lowly methylated (LM): < 25% methylation ratio) (Chi-squared test, *P* < 2.20 × 10^-16^).**


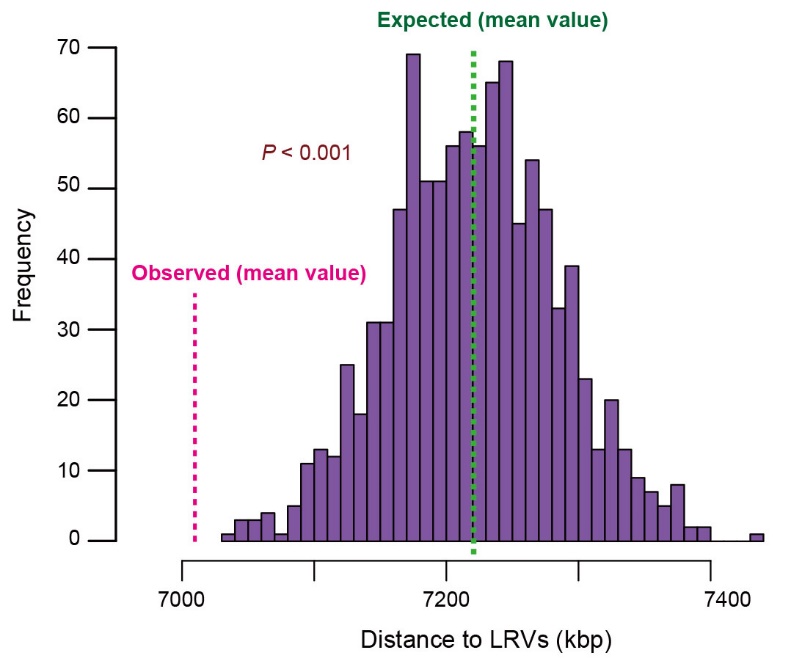


**Figure S4. The distance difference of CEN-specific hyper-DMCs from LRVs compared to that of randomly selected CpG sites (The *p* value was calculated by bootstrap test with 1000 random samplings).**


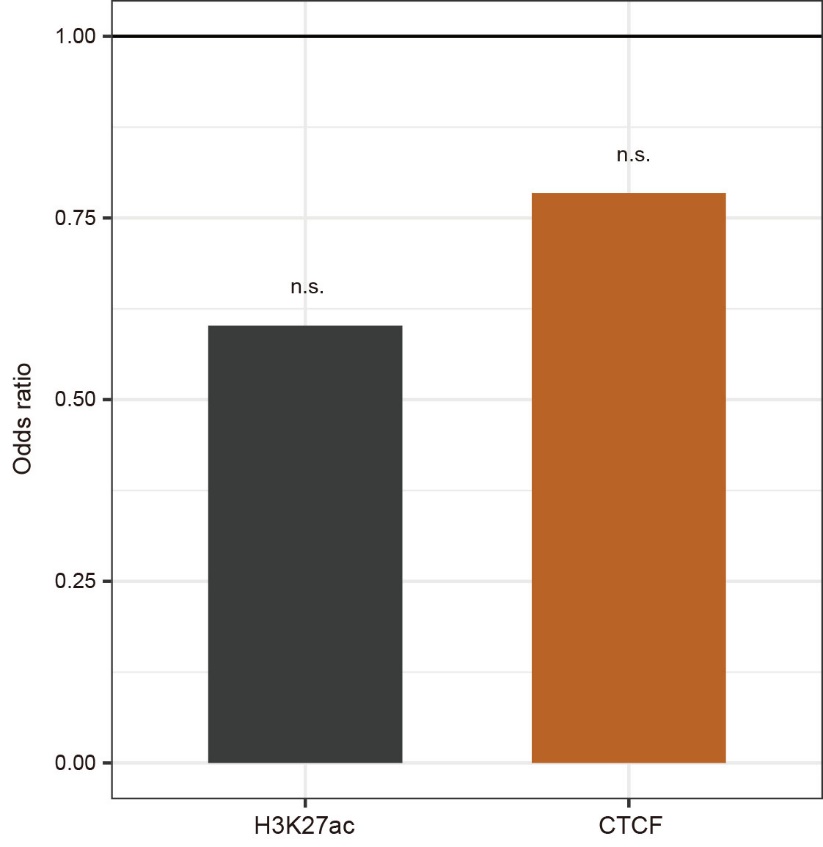


**Figure S5. Enrichment analysis for the CEN-specific hyper-DMCs in the H3K27ac and CTCF regions in GM12878 cells. (n.s., non-significant)**


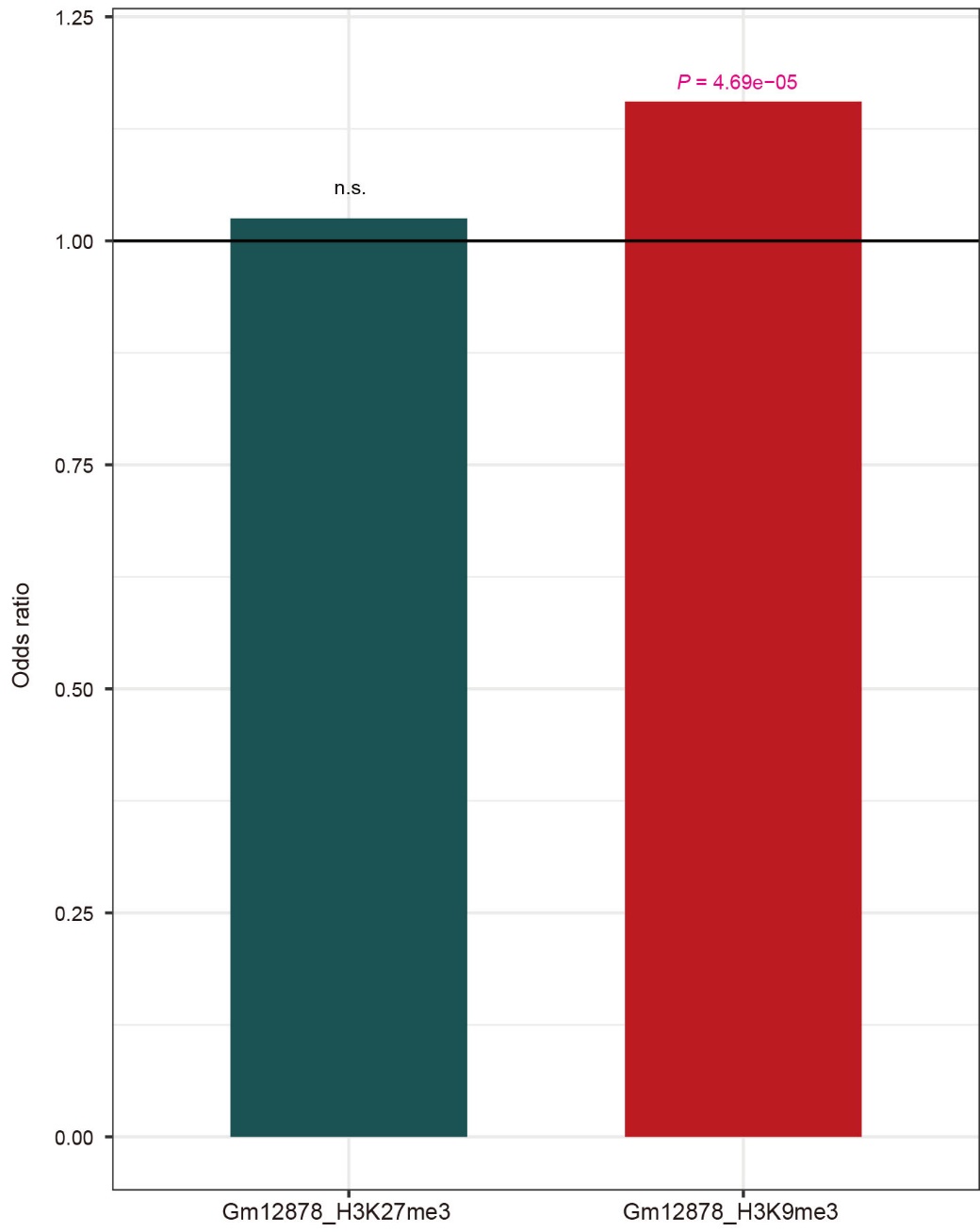


**Figure S6. Enrichment analysis for the CEN-specific hyper-DMCs (methylation difference > 20%) in the H3K9me3 and H3K27me3 regions in GM12878 cells. (n.s., non-significant)**


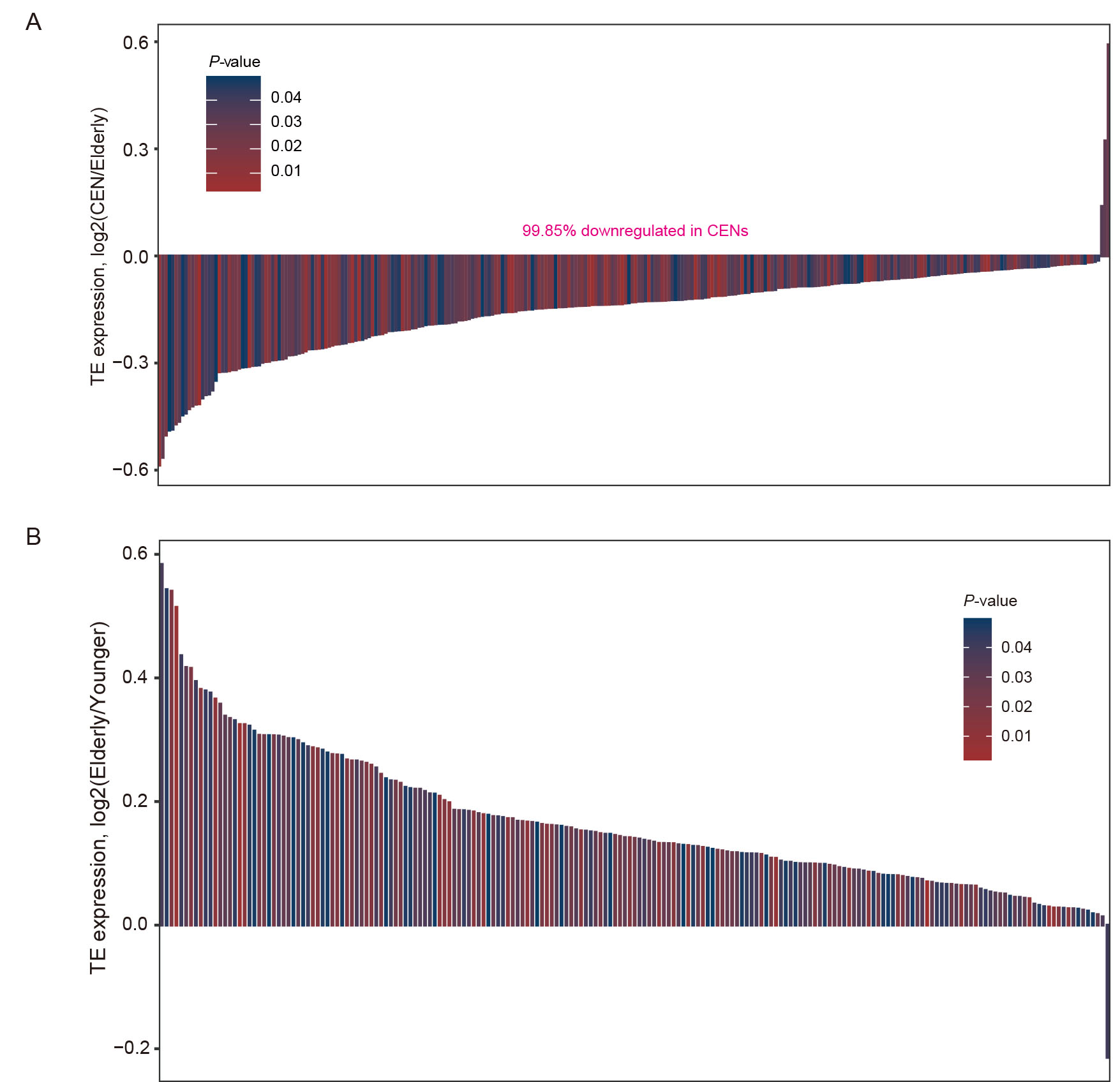


**Figure S7. Expression differences of TEs in H3K9me3 regions (GM12878) with CEN-specific hyper-DMCs between the centenarians and elderly F1SP samples (A), and between the elderly and younger F1SP samples (B).**


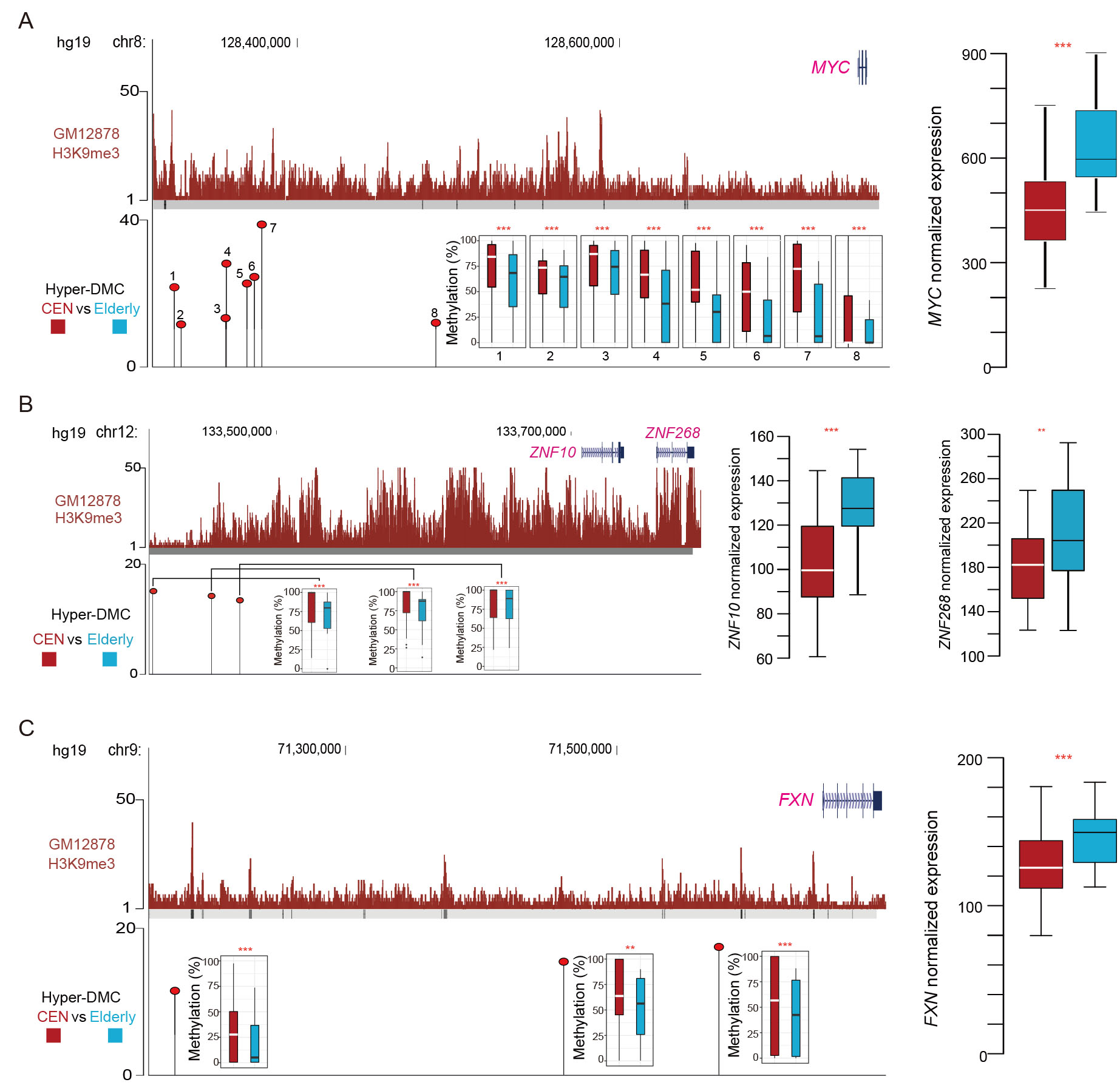


**Figure S8. Potential roles of some CEN-specific hyper-DMCs in H3K9me3 regions in human healthy aging by repressing expression of several genes, including *MYC*, *FXN*, *ZNF10* and *ZNF268*.** (A) The gene *MYC* proto-oncogene is a tumor invasion and metastasis associated regulator, whose downregulation has been reported to improve longevity and healthspan in mice [17]. The hyper-DMCs located in the nearby H3K9me3 region of *MYC* may contribute to healthy aging via suppressing its expression. (B) Previous studies have shown that the reduced expression of several ZNF genes, including *ZNF10* and *ZNF268*, has function in inhibiting cancer development [18–20], likely echoing the markedly low incidence of cancer in centenarians [21]. (C) The gene *FXN* encodes a mitochondrial protein frataxin functioning in the regulation of iron transport. Previous studies have shown that the reduced *FXN* expression can induce ferroptosis and inhibit tumor growth [22], it is likely that the hyper-DMCs in H3K9me3 regions nearby *FXN* has potential to contribute to human healthy aging by inhibiting cancer. (**, *P* < 0.01; ***, *P* < 0.001)


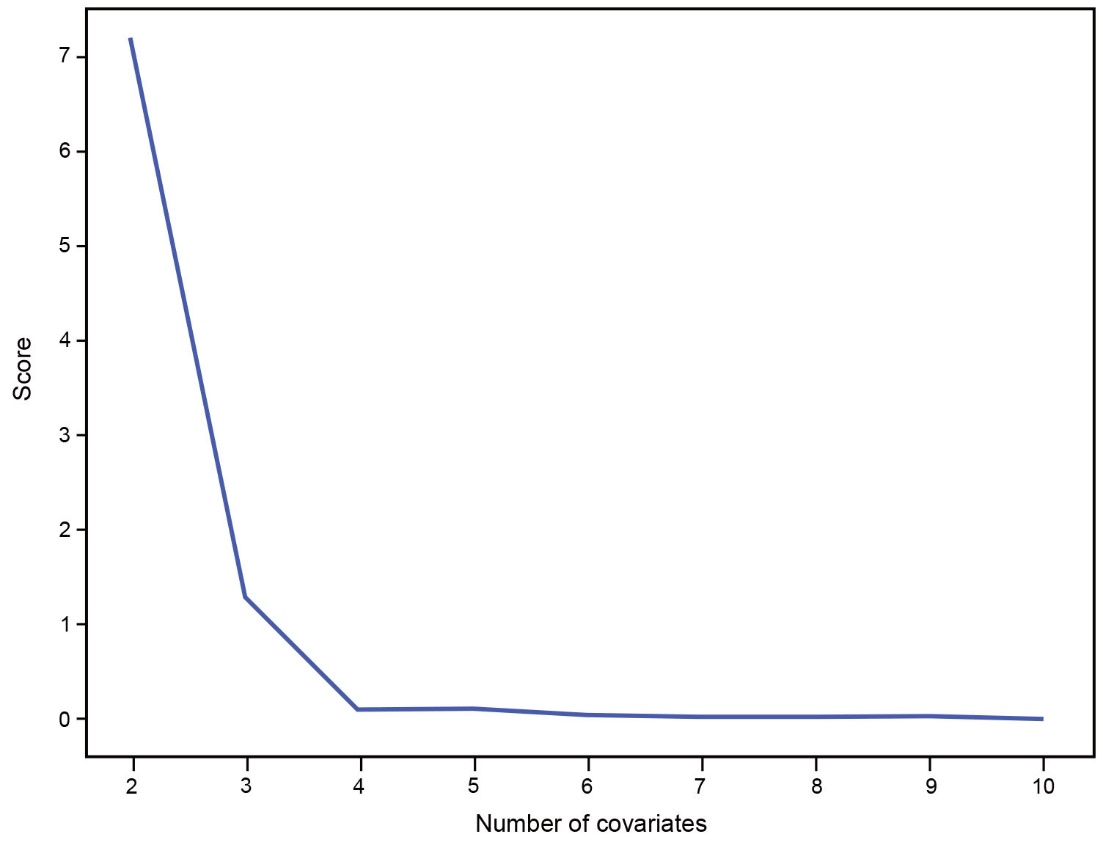


**Figure S9. Selection of the best number of covariable components in inferring cell composition.** The scores for the candidate numbers of covariable components, which was calculated by ReFACTor's built-in script. According to ReFACTor's manual, the number of covariates should be selected to be the number of high score eigenvalues, before reaching a right tail of flat scores.

**REFERENCES**

1. Xiao FH, Chen XQ, Yu Q *et al.* Transcriptome evidence reveals enhanced autophagy-lysosomal function in centenarians. *Genome Res* 2018;**28**:1601–10.

2. Krueger F, Andrews SR. Bismark: a flexible aligner and methylation caller for Bisulfite-Seq applications. *Bioinformatics* 2011;**27**:1571–2.

3. Langmead B, Salzberg SL. Fast gapped-read alignment with Bowtie 2. *Nat Methods* 2012;**9**:357–9.

4. Ritchie ME, Phipson B, Wu D *et al.* limma powers differential expression analyses for RNA-sequencing and microarray studies. *Nucleic Acids Res* 2015;**43**:e47–e47.

5. Akalin A, Kormaksson M, Li S *et al.* methylKit: a comprehensive R package for the analysis of genome-wide DNA methylation profiles. *Genome Biol* 2012;**13**:R87.

6. Rahmani E, Zaitlen N, Baran Y *et al.* Sparse PCA corrects for cell type heterogeneity in epigenome-wide association studies. *Nat Methods* 2016;**13**:443–5.

7. Zhang Y, Parmigiani G, Johnson WE. ComBat-seq: batch effect adjustment for RNA-seq count data. *NAR Genom Bioinform* 2020;**2**:lqaa078.

8. Love MI, Huber W, Anders S. Moderated estimation of fold change and dispersion for RNA-seq data with DESeq2. *Genome Biol* 2014;**15**:550.

9. Kong Y, Rose CM, Cass AA *et al.* Transposable element expression in tumors is associated with immune infiltration and increased antigenicity. *Nat Commun* 2019;**10**:5228.

10. Yu G, Wang LG, He QY. ChIPseeker: an R/Bioconductor package for ChIP peak annotation, comparison and visualization. *Bioinformatics* 2015;**31**:2382–3.

11. Lee J-H, Demarest TG, Babbar M *et al.* Cockayne syndrome group B deficiency reduces H3K9me3 chromatin remodeler SETDB1 and exacerbates cellular aging. *Nucleic Acids Res* 2019;**47**:8548–62.

12. McCauley BS, Sun L, Yu R *et al.* Altered chromatin states drive cryptic transcription in aging mammalian stem cells. *Nat Aging* 2021;**1**:684–97.

13. Ramos EM, Hoffman D, Junkins HA *et al.* Phenotype–Genotype Integrator (PheGenI): synthesizing genome-wide association study (GWAS) data with existing genomic resources. *Eur J Hum Genet* 2014;**22**:144–7.

14. Cariaso M, Lennon G. SNPedia: a wiki supporting personal genome annotation, interpretation and analysis. *Nucleic Acids Res* 2012;**40**:D1308–12.

15. Sheffield NC, Bock C. LOLA: enrichment analysis for genomic region sets and regulatory elements in R and Bioconductor. *Bioinformatics* 2016;**32**:587–9.

16. Zhou Y, Zhou B, Pache L *et al.* Metascape provides a biologist-oriented resource for the analysis of systems-level datasets. *Nat Commun* 2019;**10**:1523.

17. Hofmann JW, Zhao X, De Cecco M *et al.* Reduced expression of MYC increases longevity and enhances healthspan. *Cell* 2015;**160**:477–88.

18. Duan J, Zhen T, Liang J *et al.* The clinicopathological significance of ZNF10 in invasive ductal carcinoma of the breast. *Int J Clin Exp Pathol* 2018;**11**:2968–79.

19. Wang W, Guo M, Hu L *et al.* The Zinc Finger Protein ZNF268 Is Overexpressed in Human Cervical Cancer and Contributes to Tumorigenesis via Enhancing NF-κB Signaling*. *J Biol Chem* 2012;**287**:42856–66.

20. Severson PL, Tokar EJ, Vrba L *et al.* Coordinate H3K9 and DNA methylation silencing of ZNFs in toxicant-induced malignant transformation. *Epigenetics* 2013;**8**:1080–8.

21. Pavlidis N, Stanta G, Audisio RA. Cancer prevalence and mortality in centenarians: A systematic review. *Crit Rev Oncol Hemat* 2012;**83**:145–52.

22. Du J, Zhou Y, Li Y *et al.* Identification of Frataxin as a regulator of ferroptosis. *Redox Biol* 2020;**32**:101483.
